# Atomic-Scale Understanding of Doping Effects in BaTiO_3_ in the Presence of Water: Implications for Photocatalytic Water Splitting

**DOI:** 10.3390/ma19112336

**Published:** 2026-06-01

**Authors:** Zhadyra Ye. Zakiyeva, Ulzhan Zh. Tolegen, Talgat M. Inerbaev, Eugene Kotomin, Aisulu U. Abuova, Beksultan Akilbekov, Ayaulym Amankeldiyeva, Arailym Zhomartova, Anatoli I. Popov, Omirzak K. Abdirashev, Fatima U. Abuova

**Affiliations:** 1 Institute of Physical and Technical Sciences, L. N. Gumilyov Eurasian National University, Astana 010000, Kazakhstan; zhadyrazakiyeva@gmail.com (Z.Y.Z.); tolegen_uzh_2@enu.kz (U.Z.T.); abuova_au@enu.kz (A.U.A.); abdirashev_ok@enu.kz (O.K.A.); 2 Vernadsky Institute of Geochemistry and Analytical Chemistry, Russian Academy of Science, 119991 Moscow, Russia; 3 Institute of Solid State Physics, University of Latvia, Kengaraga 8, LV-1063 Riga, Latvia; popov@latnet.lv; 4 National Laboratory Astana, Nazarbayev University, Astana 010000, Kazakhstan; beksultan.akilbekov@nu.edu.kz (B.A.); ayaulym.amankeldiyeva@nu.edu.kz (A.A.); 5 State Key Laboratory of Drug Research, Shanghai Institute of Materia Medica, Chinese Academy of Sciences, 555 Zuchongzhi Road, Shanghai 201203, China; arailymzhomartova@simm.ac.cn

**Keywords:** photocatalytic water splitting, barium titanate, doping, atomistic study, surface-water interaction

## Abstract

The search for efficient photocatalysts for sustainable hydrogen production has driven growing interest in barium titanate (BaTiO_3_)-based materials, particularly through polymorph control, surface engineering, and nonmetal and transition-metal doping. In this work, we provide an atomic-scale understanding of structural modifications in nitrogen-, fluorine-, and rhodium-doped BaTiO_3_ using Density Functional Theory (DFT), as well as pristine and fluorine-substituted BaTiO_3_ using reactive force-field molecular dynamics (ReaxFF-MD) simulations. DFT results for pristine and doped tetragonal BaTiO_3_, as well as pristine hexagonal BaTiO_3_, reveal that nitrogen and rhodium substitutions enhance the covalent character of Ti-N and Rh-O bonds and promote the redistribution of electron density, as evidenced by noncovalent interaction (NCI) and critical point (QTAIM) analyses, whereas fluorine substitution leads to more ionic Ti-F bonding. ReaxFF-MD simulations of pristine and fluorine-substituted BaTiO_3_ in contact with water molecules demonstrate that fluorine substitution suppresses interfacial O-H bond formation and promotes ordered molecular hydration layers near titanium sites, as reflected in bond statistics and radial distribution functions. This study provides molecular insights into the role of N, F, and Rh doping in BaTiO_3_ using DFT, and the role of fluorine doping in BaTiO_3_ at the water–solid interface using ReaxFF-MD simulations, demonstrating that this integrated computational approach provides a solid basis for the rational design of next-generation materials for energy-related applications. Direct calculations of photocatalytic activity, charge transfer rates, and ferroelectric polarization effects were not performed in this work and remain important directions for future study.

## 1. Introduction

The urgent global demand for sustainable and carbon-neutral energy technologies has intensified research into photocatalytic water splitting as a viable route for clean hydrogen production and environmental remediation [1,2,3]. By mimicking natural photosynthesis, photocatalytic systems utilize solar energy to split water into hydrogen and oxygen, offering a promising pathway toward zero-emission energy cycles. However, the practical realization of efficient photocatalytic water splitting remains challenging, requiring materials that simultaneously exhibit strong light absorption, efficient charge separation and transport, chemical stability in aqueous environments, and scalability for real-world applications [4,5,6,7,8,9,10,11,12,13,14,15,16].

Among oxide-based photocatalysts, barium titanate (BaTiO_3_), a prototypical perovskite material, has attracted considerable attention due to its chemical robustness, and tunable electronic structure [14,15,16,17,18,19,20,21,22,23,24,25,26,27]. Nevertheless, pristine BaTiO_3_ is limited by its relatively wide band gap, which restricts efficient utilization of visible light and constrains its photocatalytic performance under solar irradiation [27,28,29,30,31,32].

To overcome these limitations, extensive efforts have been devoted to band structure engineering of BaTiO_3_ through surface modification and elemental doping [33,34,35,36,37,38,39,40,41,42,43]. Importantly, the photocatalytic response of doped BaTiO_3_ is highly sensitive to dopant chemistry, substitution site, and local coordination environment, underscoring the need for atomistic-level understanding. Beyond this, the interaction between water molecules and BaTiO_3_ surfaces plays an important role in governing photocatalytic efficiency [44,45,46,47,48,49,50]. Experimental and theoretical studies have shown that water can adsorb molecularly or dissociatively on BaTiO_3_ surfaces, with surface oxygen and titanium sites acting as key reactive centers.

Despite significant progress, a comprehensive atomic-scale picture of how different dopants simultaneously affect BaTiO_3_ surface structure, electronic properties, and dynamic water behavior remains incomplete [51]. Our recent comprehensive review of atomistic studies on BaTiO_3_ photocatalysts [51] identified that among the various doping strategies reported in the literature, nonmetal doping with nitrogen and fluorine, as well as transition-metal doping with rhodium, represent particularly promising yet underexplored avenues for enhancing photocatalytic water-splitting performance. Nitrogen doping has been shown to narrow the bandgap of BaTiO_3_ by introducing localized N 2p states that hybridize with O 2p orbitals [51,52,53]. Fluorine doping, by contrast, offers a distinct passivation effect due to its high electronegativity, potentially stabilizing surface terminations and modifying surface acidity without introducing deep-level recombination centers [51,52,53,54]. Rhodium doping, as a transition-metal substitution at Ti sites, can introduce intermediate oxidation states (Rh^3+^/Rh^4+^) [51,52,53,54,55,56]. While other dopants (e.g., C, S, Se, La, Mo, Ir) have been studied to varying degrees, the distinct and complementary mechanisms of N, F, and Rh, spanning bandgap engineering and surface passivation, make them scientifically compelling candidates for systematic comparative investigation. Most previous computational studies have focused either on static electronic properties under vacuum conditions or on isolated dopant effects, often neglecting the complex and dynamic nature of the solid–liquid interface [56,57,58,59,60,61,62,63].

In this work, we address these gaps by combining Density Functional Theory (DFT) calculations with reactive force-field molecular dynamics (ReaxFF-MD) simulations to investigate doped BaTiO_3_ surfaces. The molecular dynamic simulations reliably reflect the structural and thermodynamic baseline established by the static DFT analyses. Herein, dopant incorporation was primarily considered at Ti and O lattice sites. While A-site (Ba-site) doping can also influence material properties, its effects are generally more indirect and were therefore beyond the scope of the present study. In addition to tetragonal BaTiO_3_, the hexagonal phase was considered as a reference to evaluate intrinsic phase-dependent differences in structural and electronic properties. This comparison allows for clearer separation of the effects arising from crystal phase and those induced by doping, thereby providing a more comprehensive understanding of structure–property relationships.

The primary scope of the present study is therefore to provide atomic-scale insights into molecular-level interactions and interfacial dynamics between doped BaTiO_3_ surfaces and water, rather than to compute bulk electronic properties such as band structures, density of states, or band edge positions. While the latter are undoubtedly important for photocatalytic performance and have been extensively reported for pristine and Rh-doped BaTiO_3_ in our previous work [64] and reviewed comprehensively by [51], the present work focuses uniquely on the interplay of local bonding topology in nitrogen-, fluorine-, and rhodium-doped BaTiO_3_ using DFT, and dynamic water–surface interactions in pristine and fluorine-substituted BaTiO_3_ using ReaxFF-MD simulations. Importantly, the present study does not directly calculate photocatalytic activity metrics (e.g., hydrogen evolution rates, quantum efficiencies, or charge carrier mobilities). Rather, our focus is on static electronic structure modifications induced by doping (DFT) and dynamic water–surface interactions (ReaxFF-MD) as foundational inputs for future photocatalytic modeling.

## 2. Materials and Methods

### 2.1. System of Interest

**Figure 1:**
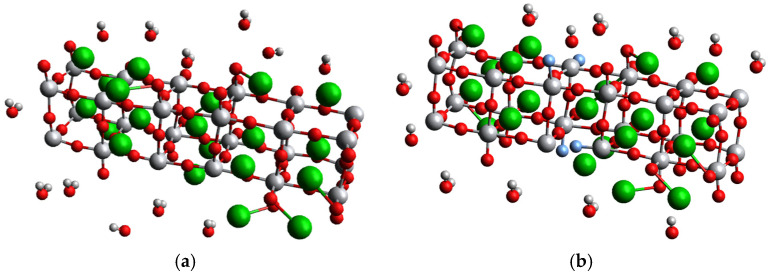
The 3D structures of aqueous barium titanate systems: (**a**) pristine tetragonal BaTiO_3_ and (**b**) tetragonal BaTiO_3_ featuring four oxygen atom substitutions by fluorine (higher local dopant density than the DFT single-substitution models; see Section 2.3 for discussion of this methodological distinction). Atom colors are assigned as follows: Ba (green), Ti (grey), O (red), H (light grey), and F (blue).

**Figure 2:**
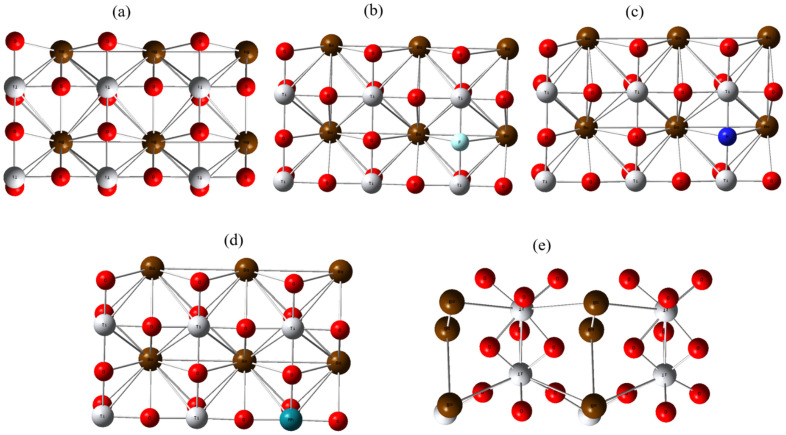
The 3D optimized structures of (**a**) tetragonal BaTiO_3_, (**b**) tetragonal BaTiO_3_ doped with fluoride, (**c**) tetragonal BaTiO_3_ doped with nitrogen, (**d**) tetragonal BaTiO_3_ doped with rhodium, and (**e**) hexagonal BaTiO_3_. Color key: dark orange (barium); grey (titanium); red (oxygen); white-green (fluoride); blue (nitrogen); dark green (rhodium). DFT calculations were performed using a 2 × 2 × 2 supercell.

The BaTiO_3_ theoretical model used in this work was adopted from the previously optimized and validated structure reported by [8,14,34,64]. Based on this reference model, five BaTiO_3_-based systems were constructed for DFT calculations: (i) pristine tetragonal BaTiO_3_, (ii) F-doped tetragonal BaTiO_3_, (iii) N-doped tetragonal BaTiO_3_, (iv) Rh-doped tetragonal BaTiO_3_, and (v) pristine hexagonal BaTiO_3_ (Figure 1 and Figure 2).

For surface-based simulations, a TiO_2_-terminated (001) slab was employed, as this surface is the most energetically stable and widely reported as the most relevant for photocatalytic water-splitting reactions, consistent with previous studies [64].

A 2 × 2 × 2 supercell (8 formula units of BaTiO_3_, 40 atoms total) was constructed from the optimized tetragonal unit cell (a = b = 3.994 Å, c = 4.034 Å). A single substitution per supercell was used for all dopants, corresponding to the following concentrations: (i) N-doping (1 O → N): 0.75 wt% (4.2 at% of the O sublattice), (ii) F-doping (1 O → F): 1.0 wt% (4.2 at% of the O sublattice), and (iii) Rh-doping (1 Ti → Rh): 5.4 wt% (12.5 at% of the Ti sublattice). This concentration has been successfully employed in prior computational studies of doped perovskite photocatalysts and provides a reasonable balance between computational tractability and experimental relevance [35,36,37,38,39,40,52,53,54].

Moreover, we note an important methodological distinction between the DFT and ReaxFF-MD models for fluorine-doped BaTiO_3_. The DFT calculations employed a single oxygen substitution per 2 × 2 × 2 supercell, whereas the ReaxFF-MD simulations utilized four oxygen substitutions in a comparable supercell to enhance statistical sampling of interfacial phenomena within the computationally demanding explicit-water environment. This difference in dopant density means that the observed suppression of O–H bond formation in the ReaxFF-MD trajectories reflects the combined effects of fluorine chemistry and a higher local density of substitution sites. The present ReaxFF-MD results should therefore be interpreted as demonstrating a trend (suppression of surface hydroxylation upon fluorine incorporation) rather than providing a direct quantitative comparison with the DFT single-substitution case. Future simulations using systematically varied dopant concentrations are needed to deconvolute chemical versus concentration effects.

For ReaxFF-MD simulations, a TiO_2_-terminated (001) slab was cleaved from the optimized tetragonal BaTiO_3_ bulk structure. A 4 × 4 in-plane supercell (relative to the primitive 1 × 1 surface cell) was constructed, yielding lateral dimensions of approximately 15.98 × 15.98 Å^2^. The slab contained 6 atomic layers (thickness ~12 Å), with the bottom two layers constrained to bulk positions to mimic a semi-infinite substrate. A 15 Å vacuum gap was added above the surface, followed by 880 explicit water molecules (density ≈ 1.0 g/cm^3^, water layer thickness ≈ 25 Å). The total simulation cell dimensions were Lx = 15.98 Å, Ly = 15.98 Å, Lz = 52.00 Å.

To account for dynamic water–surface interactions under realistic conditions, ReaxFF-MD simulations were conducted for pristine and F-doped tetragonal BaTiO_3_ in the presence of explicit water molecules.

Quantitative water adsorption energies and free energy profiles for molecular versus dissociative adsorption are not reported in this work, as the present DFT calculations are cluster-based (Gaussian16) rather than plane-wave (VASP), and the ReaxFF-MD simulations did not incorporate enhanced sampling techniques (e.g., PLUMED). Such calculations are planned for future studies using machine learning potentials interfaced with VASP and LAMMPS.

Moreover, because the present DFT calculations are performed using cluster-based Gaussian16 (rather than periodic plane-wave VASP), we do not report bulk band structures, DOS/PDOS, or band edge positions. These properties for pristine and Rh-doped BaTiO_3_ have been reported previously by our group [64], and a broader review of electronic structure calculations for doped BaTiO_3_ photocatalysts is available in Abuova et al. [51] work.

### 2.2. DFT Calculations

All DFT models investigated in this study were previously fully optimized using the Vienna Ab initio Simulation Package version 5.4.4 (VASP 5.4.4) [64], as reported in our earlier works and validated against structures from the Materials Project database. To verify the dynamical stability of the optimized structures, frequency calculations were performed using the Gaussian16 package (version 16) [65] and visualized with GaussView 6.0 [66]. All vibrational frequencies were confirmed to be converged, with no imaginary modes observed.

All DFT calculations in this work were carried out using the screened hybrid HSEH1PBE functional, which provides an improved description of electronic exchange-correlation effects compared to standard GGA functionals and is widely used for transition-metal oxide systems. The def2-TZVP basis set was employed for all atoms, offering a reliable balance between computational cost and accuracy for structural and electronic analyses. The initial geometries of all BaTiO_3_-based systems were adopted from previously optimized and validated structures reported by Inerbaev et al. [64]. In the present study, these structures were further subjected to vibrational frequency calculations to confirm their dynamical stability; all systems exhibited fully converged frequencies with no imaginary modes, indicating true local minima on the potential energy surface.

For pristine, F-doped, and N-doped systems, a spin-restricted formalism was applied. In contrast, Rh-doped systems were treated using a spin-unrestricted approach to accurately capture the open-shell electronic configuration of the transition-metal dopant. Tight self-consistent field convergence criteria and Fermi smearing were applied to ensure numerical stability and reliable electronic structure characterization.

DFT calculation outputs were subsequently employed for optimized structural analysis, Mulliken charge analysis, RDG, NCI analysis, and CP analysis using the Multiwfn (version 3.7) software [67] and VMD (version 1.9.1) software [68]. Mulliken charge analysis is employed here as a qualitative tool for comparing relative charge distributions across different dopant systems. We caution that absolute charge values are basis-set-dependent and should not be overinterpreted as formal oxidation states. The latter are more reliably determined from complementary analyses such as Bader charges or X-ray absorption near-edge structure (XANES) simulations, which are beyond the scope of the present work.

### 2.3. Reactive Molecular Dynamics Simulations

All ReaxFF-MD simulations were performed using LAMMPS (version 23 June 2022) [69]. The key simulation parameters are detailed below to ensure reproducibility. Two simulation cells were considered: a tetragonal BaTiO_3_ slab in contact with liquid water (System 1), and an analogous slab in which four lattice oxygen sites were replaced by fluorine (System 2, denoted “F-substituted BaTiO_3_”). Figure 1 shows representative all-atom reactive molecular dynamics snapshots for (a) tetragonal BaTiO_3_ in water and (b) the model with four oxygen sites substituted by fluorine in water. The interatomic interactions were defined via pair_style reaxff, and atomic charges were dynamically updated using the fix qeq/reaxff equilibration scheme. We employed the barium titanate parameterization developed by Akbarian et al. (2021) [70].

Prior to production MD, the system was energy-minimized using a conjugate gradient algorithm with convergence criteria of 1.0 × 10^−8^ (energy tolerance) and 1.0 × 10^−10^ (force tolerance). A Verlet neighbor list with a cutoff distance of 2.0 Å beyond the ReaxFF interaction cutoff was employed. The neighbor list was rebuilt every timestep (neigh_modify delay 0 every 1 check yes) to ensure accuracy given the reactive nature of the force field. The velocity-Verlet integration algorithm was used with a fixed timestep of 1.0 fs, selected to resolve high-frequency O–H vibrational modes (characteristic period ~10 fs). Temperature was maintained at 300 K using a Nosé–Hoover thermostat with a damping parameter of 100.0 fs. A total of 10,000,000 MD steps were performed, corresponding to 10 ns of simulation time. The trajectory was saved every 10,000 steps (10 ps) in LAMMPS custom dump format. The fix qeq/reaxff charge equilibration scheme was applied every timestep with a convergence tolerance of 1.0 × 10^−6^ e. Three-dimensional periodic boundary conditions were applied. The bottom two atomic layers of the BaTiO_3_ slab were fixed using the setforce command (zeroing forces in all directions), and their velocities were set to zero to mimic a semi-infinite bulk substrate. A relaxation protocol consisting of energy minimization followed by NVT dynamics at 300 K controlled by Nosé–Hoover thermostat was used to obtain equilibrated interfacial structures for a total simulation time of 10 ns.

Structural and dynamical properties were analyzed using time-averaged trajectories over the equilibrated simulation window. Key analysis metrics included the evolution of atomic charges (via ReaxFF charge equilibration), RDFs to characterize interfacial structure, and qualitative assessment of water adsorption and dissociation events at the surface. All analyses were performed using standard LAMMPS post-processing tools and in-house scripts to ensure reproducibility (see Supplementary Materials). 

It should be noted that ReaxFF-MD simulations for N- and Rh-doped BaTiO_3_ could not be performed due to the lack of reliable force field parameters for nitrogen and rhodium within the BaTiO_3_ lattice environment; attempts for N-doping led to severe instabilities, while Rh parameters are entirely absent in the current force field [70]. Moreover, this ReaxFF approach has demonstrated strong performance in modeling complex interfacial phenomena, including polymer electrolyte membrane fuel cell, and wastewater treatment applications [71,72]. Future work will focus on computing water adsorption energies and reaction free energies for different dopants and surface terminations using plane-wave DFT and enhanced sampling molecular dynamics. To analyze the electronic state of the system, average per-atom charges for Ba, Ti, lattice oxygen, and water species were monitored using group-averaged variables.

Bonds were identified based on distance cutoffs determined from the first minimum of the radial distribution function (RDF) for each atomic pair (see Supplementary Materials), calculated over the equilibrated trajectory (last 8 ns). A bond was considered ‘formed’ when the interatomic distance remained below the cutoff for at least three consecutive timesteps (3 fs) to exclude transient fluctuations. RDFs were averaged over the production trajectory (8 ns) and over all symmetry-equivalent atomic pairs.

## 3. Results and Discussion

### 3.1. DFT Calculation Results

#### 3.1.1. Optimized Structures

Figure 2 and Table 1 present the three-dimensional geometries and optimized structural parameters of pristine and doped BaTiO_3_ systems, highlighting the structural responses of the lattice to anion (N, F) and cation (Rh) substitutions. All DFT-optimized structures are dynamically stable, with no imaginary vibrational modes observed.

Pristine tetragonal BaTiO_3_ (Figure 2a, Table 1) preserves the characteristic perovskite framework, where Ti atoms occupy the centers of slightly distorted TiO_6_ octahedra and Ba atoms are coordinated by surrounding oxygen atoms. The calculated Ti-O bonds average 1.97 Å with O-Ti-O angles of 88.5–91.5°, consistent with experimental values and confirming the slight tetragonal distortion associated with ferroelectric behavior [64].

Fluorine-doped tetragonal BaTiO_3_ (Figure 2b, Table 1) involves substitution of lattice oxygen by fluorine. Due to the similar ionic radii of F^−^ and O^2−^, the overall perovskite framework remains intact. The Ti-F bond shortens to 1.94 Å with minimal angular deviation (F-Ti-O: 86.5–93.5°), reflecting ionic passivation and only localized distortions.

Nitrogen-doped tetragonal BaTiO_3_ (Figure 2c, Table 1) exhibits more pronounced local distortions compared to fluorine substitution. The lower electronegativity and different bonding characteristics of nitrogen result in an elongated Ti-N bond (2.09 Å) and angular distortion (N-Ti-O: 84.5–95.5°), indicating increased covalent character and asymmetric coordination.

Rhodium-doped tetragonal BaTiO_3_ (Figure 2d, Table 1) substitutes a Ti atom with Rh. The difference in ionic size and valence between Rh and Ti induces noticeable lattice relaxation, with Rh-O bonds lengthened to 2.01 Å and octahedral angles expanded to 86.5–93.5°. These features are consistent with intermediate Rh^3+^/Rh^4+^ oxidation states and strong dopant–lattice coupling.

Hexagonal BaTiO_3_ (Figure 2e, Table 1) exhibits a distinct stacking arrangement compared to the tetragonal phase, with alternating face-sharing and corner-sharing TiO_6_ octahedra reflected in O-Ti-O angles ranging from 85.0° to 95.0°. This structural diversity provides an alternative platform for tuning surface and electronic properties.

#### 3.1.2. Noncovalent Interactions

**Figure 3:**
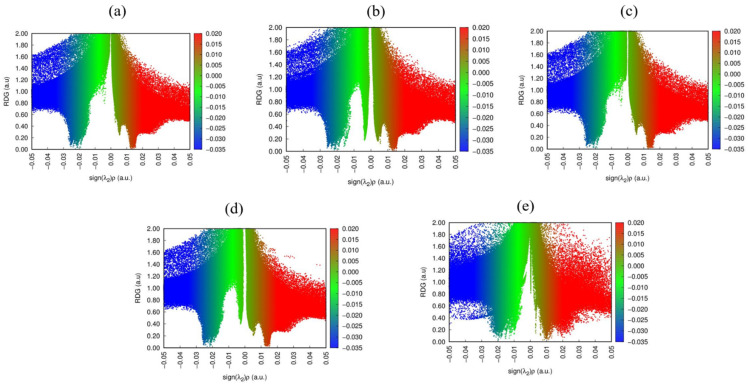
RDG of (**a**) tetragonal BaTiO_3_, (**b**) tetragonal BaTiO_3_ doped with fluoride, (**c**) tetragonal BaTiO_3_ doped with nitrogen, (**d**) tetragonal BaTiO_3_ doped with rhodium, and (**e**) hexagonal BaTiO_3_.

The reduced density gradient (RDG) analysis provides valuable insight into the nature and strength of noncovalent interactions (NCIs) present in pristine and doped BaTiO_3_ structures (Figure 3). For pristine tetragonal BaTiO_3_ (Figure 3a), the RDG isosurfaces primarily indicate weak van der Waals interactions distributed around the Ti-O octahedra and Ba-O coordination environment. These interactions reflect the intrinsic ionic-covalent balance of the perovskite lattice and serve as a reference for evaluating dopant-induced modifications.

Upon fluorine substitution (Figure 3b), noticeable changes in the RDG features emerge near the substituted sites. The appearance of localized regions with altered RDG signatures suggests modification of weak interactions surrounding Ti-F bonds compared to Ti-O bonds. This reflects fluorine’s higher electronegativity, which redistributes electron density and weakens local attractive interactions.

In the nitrogen-doped BaTiO_3_ system (Figure 3c), the RDG plots show more pronounced features associated with stronger attractive interactions. Nitrogen substitution introduces localized electronic perturbations that enhance directional interactions between Ti and neighboring anions. These features are consistent with partial covalent character in Ti-N bonding.

The rhodium-doped structure (Figure 3d) exhibits distinct RDG signatures around the Rh-centered octahedron. Stronger attractive interactions are observed between Rh and surrounding oxygen atoms, indicating significant metal–oxygen hybridization.

Hexagonal BaTiO_3_ (Figure 3e) displays a more heterogeneous RDG distribution compared to the tetragonal phase, reflecting its distinct stacking sequence and coordination environments. The presence of varied weak and intermediate interactions highlights the structural flexibility of the hexagonal polymorph.

**Figure 4:**
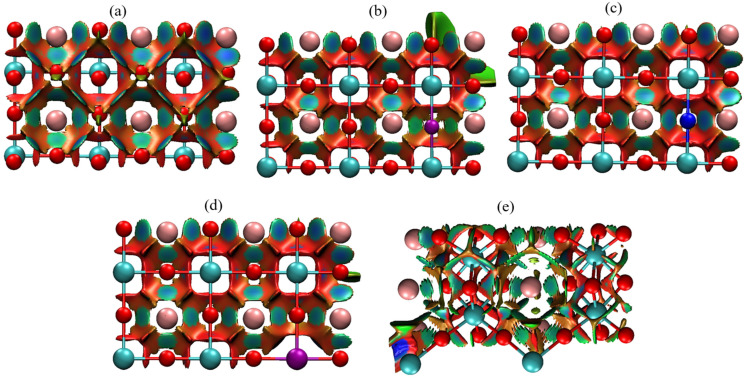
NCIs of (**a**) tetragonal BaTiO_3_, (**b**) tetragonal BaTiO_3_ doped with fluoride, (**c**) tetragonal BaTiO_3_ doped with nitrogen, (**d**) tetragonal BaTiO_3_ doped with rhodium, and (**e**) hexagonal BaTiO_3_. Color key of atoms: dark orange (barium); grey (titanium); red (oxygen); light violet (fluoride); blue (nitrogen); dark violet (rhodium). Color scheme of NCI (isosurface): blue (strong attraction), green (van der Waals interaction), and red (strong repulsion). Various colored lines are bonds of atoms. DFT calculations were performed using a 2 × 2 × 2 supercell.

The NCI visualization further elucidates the spatial distribution and nature of weak interactions governing the stability and reactivity of pristine and doped BaTiO_3_ systems (Figure 4). In pristine tetragonal BaTiO_3_ (Figure 4a), NCI isosurfaces are mainly associated with weak dispersive interactions between Ba and surrounding oxygen atoms, consistent with the predominantly ionic Ba-O coordination. These interactions contribute to lattice stabilization but do not introduce strong directional bonding features.

Fluorine-doped BaTiO_3_ (Figure 4b) exhibits a redistribution of NCI regions near the substituted fluorine atoms. The reduced intensity of attractive NCI features around these sites suggests weakened hydrogen-bonding capability. This observation supports the role of fluorine as a passivating dopant that stabilizes the lattice while limiting chemically active noncovalent interactions.

In the nitrogen-doped structure (Figure 4c), enhanced NCI features are observed near Ti-N linkages, indicating stronger attractive interactions compared to the fluorine-doped case. These regions suggest increased electronic localization and potential active sites for molecular adsorption. The presence of stronger noncovalent interactions aligns with nitrogen’s ability to modify local electronic structure and promote surface reactivity.

The rhodium-doped BaTiO_3_ system (Figure 4d) displays the most pronounced NCI features, particularly around the Rh-O coordination environment. The dense and localized attractive regions indicate strong dopant–lattice coupling.

In contrast, hexagonal BaTiO_3_ (Figure 4e) shows a broader distribution of NCI features, reflecting its more complex coordination network. The coexistence of weak and moderate interactions suggests a structurally adaptable surface capable of accommodating adsorbates through multiple interaction modes.

The NCI analysis highlights how dopant chemistry and crystal phase synergistically tune weak interactions, providing an atomistic foundation for understanding the photocatalytic behavior of BaTiO_3_-based materials.

#### 3.1.3. Critical Points

**Figure 5:**
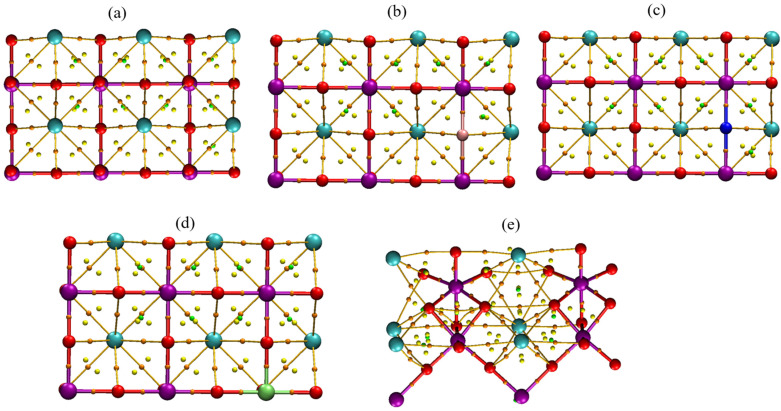
CPs of (**a**) tetragonal BaTiO_3_, (**b**) tetragonal BaTiO_3_ doped with fluoride, (**c**) tetragonal BaTiO_3_ doped with nitrogen, (**d**) tetragonal BaTiO_3_ doped with rhodium, and (**e**) hexagonal BaTiO_3_. Color key of atoms: grey (barium); violet (titanium); red (oxygen); orange (fluoride); blue (nitrogen); light green (rhodium). The yellow indicate bond critical point (BCP) paths between atoms. DFT calculations were performed using a 2 × 2 × 2 supercell.

Critical point (CP) analysis based on the quantum theory of atoms in molecules (QTAIM) provides quantitative insight into the bonding characteristics and electron density distribution in pristine and doped BaTiO_3_ systems (Figure 5, Table 2).

Herein, Figure 5 presents the distribution of CPs for pristine and doped BaTiO_3_ systems, with quantitative BCP descriptors summarized in Table 2.

In pristine tetragonal BaTiO_3_ (Figure 5a, Table 2), BCPs are clearly observed along Ti-O and Ba-O interactions. Ti-O BCPs exhibit ρ(r) = 0.143–0.182 a.u. with negative Laplacian values (−0.303 × 10^6^ to −0.399 × 10^6^ a.u.), indicating partial covalent character superimposed on an ionic framework. Ba-O interactions show lower electron density (ρ(r) = 0.012–0.019 a.u.) and positive Laplacian values (+0.045 to +0.092 a.u.), consistent with predominantly ionic bonding.

In the fluorine-doped BaTiO_3_ structure (Figure 5b, Table 2), replacement of oxygen by fluorine leads to distinct changes in the local topological features. Ti-F BCPs display reduced electron density (ρ(r) = 0.112–0.146 a.u.) and less negative Laplacian values (−0.241 × 10^6^ to −0.319 × 10^6^ a.u.) compared to pristine Ti-O bonds. This reflects the stronger electronegativity of fluorine and a more polarized, ionic bonding nature. Additionally, subtle shifts in nearby BCPs suggest a charge density redistribution within the TiO_6_ octahedra.

Nitrogen doping (Figure 5c, Table 2) introduces more pronounced modifications in the CP topology. Ti-N BCPs exhibit increased electron density (ρ(r) = 0.168–0.196 a.u.) and more negative Laplacian values (−0.352 × 10^6^ to −0.421 × 10^6^ a.u.) relative to pristine Ti-O, indicating enhanced covalent character. This behavior supports the formation of electronically active sites and is consistent with the RDG and NCI analyses showing stronger local interactions.

Rhodium substitution at the Ti site (Figure 5d, Table 2) produces significant topological changes in the electron density distribution. Rh-O BCPs exhibit elevated electron density (ρ(r) = 0.159–0.189 a.u.) with strongly negative Laplacian values (−0.341 × 10^6^ to −0.408 × 10^6^ a.u.), suggesting strong metal-oxygen hybridization. These features reflect substantial dopant-lattice coupling, which can introduce localized electronic states and enhance catalytic activity.

Finally, the hexagonal BaTiO_3_ structure (Figure 5e, Table 2) exhibits a more complex network of critical points due to its distinct stacking and coordination environment. Two distinct Ti-O BCP types are observed: face-sharing octahedra show weaker bonding (ρ(r) = 0.099–0.136 a.u., ∇^2^ρ(r) = −0.210 × 10^6^ to −0.289 × 10^6^ a.u.), while corner-sharing octahedra exhibit stronger bonding (ρ(r) = 0.145–0.184 a.u., ∇^2^ρ(r) = −0.311 × 10^6^ to −0.402 × 10^6^ a.u.) comparable to tetragonal BaTiO_3_. Ba-O interactions (ρ(r) = 0.010–0.016 a.u., positive Laplacian) remain ionic. The coexistence of multiple BCP types highlights structural heterogeneity and varied bonding interactions, underscoring the flexibility of the hexagonal phase for tuning photocatalytic properties.

#### 3.1.4. Mulliken Charges

**Figure 6:**
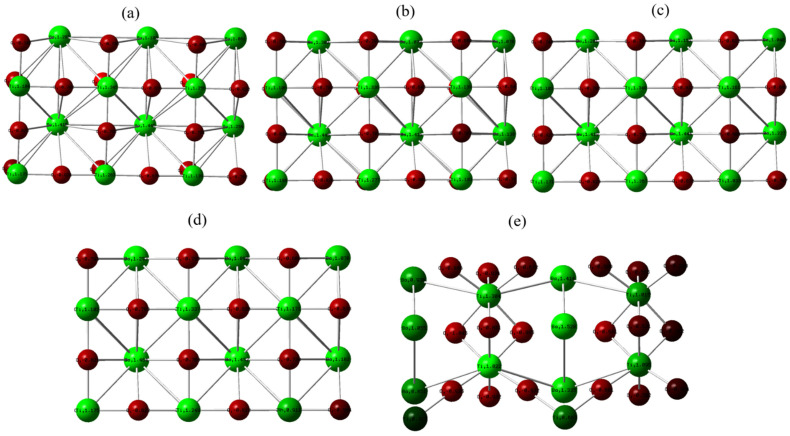
Mulliken charges of (**a**) tetragonal BaTiO_3_, (**b**) tetragonal BaTiO_3_ doped with fluoride, (**c**) tetragonal BaTiO_3_ doped with nitrogen, (**d**) tetragonal BaTiO_3_ doped with rhodium, and (**e**) hexagonal BaTiO_3_. Color key: color bar ranges from dark red to light green indicate charge from negative to positive, charge range from (−1.82 to + 1.82 e). DFT calculations were performed using a 2 × 2 × 2 supercell.

Figure 6 presents Mulliken charge distributions for pristine and doped BaTiO_3_ systems (charge range: −1.82 to +1.82 e). While Mulliken charges are basis-set dependent and provide only qualitative or semi-quantitative trends in charge redistribution rather than absolute oxidation states, they offer useful insights into relative dopant-induced electronic perturbations.

In pristine tetragonal BaTiO_3_, Ba atoms exhibit charges of approximately +1.52 to +1.65 e, Ti shows +1.35 to +1.48 e, while O atoms range from −1.05 to −1.18 e. These values reflect mixed ionic-covalent bonding, though the magnitude of charge transfer should be interpreted with caution due to the well-known sensitivity of Mulliken populations to the chosen basis set.

Replacement of O^2−^ by F^−^ introduces a formal charge deficit, as F^−^ carries one less negative charge than O^2−^. The calculated Mulliken charge of F is approximately −0.95 to −1.10 e, with adjacent Ti showing reduced positive charge (+1.20 to +1.32 e). This reduction suggests partial electron withdrawal from neighboring Ti, which may indicate a tendency toward Ti^4+^ → Ti^3+^ reduction as a charge compensation mechanism. However, alternative compensation pathways (e.g., oxygen vacancy formation) cannot be excluded based solely on Mulliken populations.

Substituting O^2−^ with N^3−^ introduces an extra negative charge, requiring either oxidation of neighboring cations or hole formation. The calculated N charge is approximately −0.85 to −1.00 e, with neighboring Ti exhibiting enhanced positive charge (+1.45 to +1.58 e) relative to pristine Ti (+1.35 to +1.48 e). This increase may indicate a shift toward higher effective Ti oxidation states (e.g., Ti^4+^ → Ti^5+^) or, more likely, increased covalent character with charge delocalization rather than strictly localized oxidation. The elevated charge on Ti is consistent with the enhanced Ti–N covalency observed in QTAIM analysis (Table 2, Figure 5c).

Substituting Ti^4+^ with Rh yields calculated Mulliken charges of approximately +1.20 to +1.35 e for Rh. This value lies between the formal limits of Rh^3+^ (+3) and Rh^4+^ (+4), indicating a mixed-valence character rather than a fixed oxidation state. Such intermediate Mulliken populations are typical for transition metals in oxide matrices and suggest that Rh adopts a superposition or resonance between Rh^3+^ and Rh^4+^ configurations, with the surrounding O atoms showing more negative charge (−1.25 to −1.38 e). This charge redistribution is consistent with significant Rh–O hybridization (Table 2, Figure 5d) and implies that Rh can flexibly accommodate charge transfer during catalytic cycles (e.g., Rh^3+^ ↔ Rh^4+^), which is favorable for oxygen evolution reaction activity. The partial charge on Rh should not be misinterpreted as a precise oxidation state but rather as an indicator of significant covalent character and charge delocalization between Rh and its neighboring oxygen ligands.

The hexagonal phase displays a broader charge distribution compared to its tetragonal counterpart, with Ba ranging from +1.45 to +1.70 e, Ti from +1.30 to +1.52 e, and O from −1.00 to −1.25 e. This heterogeneity reflects the presence of two distinct Ti–O connectivity motifs (face-sharing and corner-sharing TiO_6_ octahedra) and highlights the structural flexibility of the hexagonal polymorph.

It must be emphasized that Mulliken charges are basis-set-dependent and do not correspond to measurable physical quantities such as formal oxidation states. The values reported here serve primarily as a qualitative guide to relative charge redistribution trends induced by doping and should be interpreted alongside complementary QTAIM descriptors (Table 2) and experimental observations.

### 3.2. Reactive All-Atom Molecular Dynamics Simulation Results

#### 3.2.1. Bond Statistics and Surface Interactions

**Figure 7:**
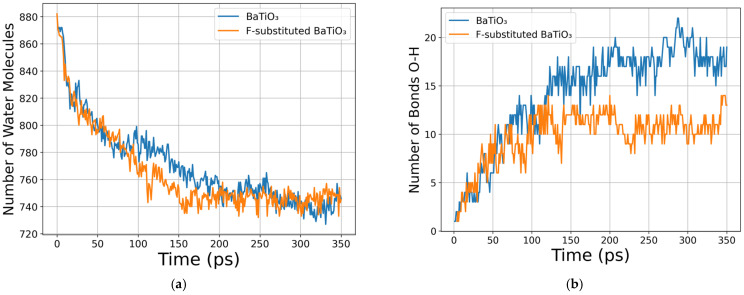

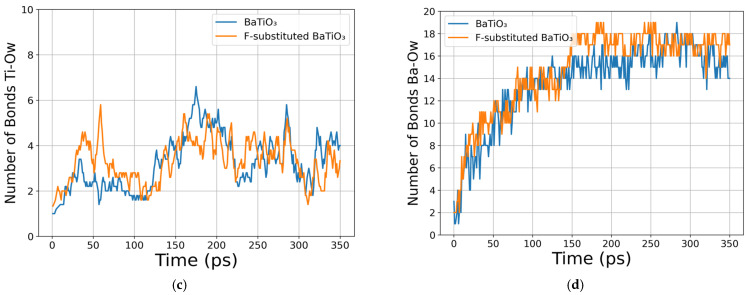
Evolution of bond statistics and surface interactions throughout the simulation. (**a**) Count of free water molecules. (**b**) Number of O–H bonds for pristine BaTiO_3_ and the F-substituted model. (**c**) Ti–Ow coordination numbers. (**d**) Ba-Ow coordination numbers.

Bond statistics and surface interaction analyses reveal the progressive formation of interfacial species and dynamic water–surface associations throughout the reactive molecular dynamics simulations. In both pristine and fluorine-substituted BaTiO_3_ systems, the number of free water molecules decreases steadily over time, dropping from approximately 880 at the start of the simulation to about 740–755 at equilibrium (Figure 7a). This reduction indicates net water consumption at the interface, which can be attributed to water adsorption, dissociation, and the formation of surface-bound hydroxyl species, as well as possible speciation changes driven by surface reactivity.

A pronounced difference between the two systems is observed in the evolution of O-H bond formation. For pristine BaTiO_3_, the number of O-H bonds increases significantly, reaching values in the range of approximately 16–21 by the end of the simulation (Figure 7b). This behavior reflects enhanced water dissociation and proton transfer processes at the oxide surface. In contrast, the fluorine-substituted BaTiO_3_ model exhibits substantially lower O-H bond numbers, stabilizing between approximately 9 and 13. This suppression of hydroxylation suggests that fluorine substitution modifies the local electronic environment and surface acidity, thereby hindering proton transfer and limiting extensive surface hydroxyl formation.

Analysis of Ti-Ow (water oxygen) coordination further supports the presence of transient adsorption events. The number of Ti-Ow bonds fluctuates between roughly 2 and 6 over the course of the simulation (Figure 7c), indicating reversible water adsorption at exposed titanium sites rather than permanent coordination. Meanwhile, Ba–Ow coordination shows a monotonic increase from near-zero values to a stable plateau of approximately 15–18 bonds (Figure 7d). Notably, the fluorine-substituted system displays a slightly higher average Ba–Ow coordination, suggesting enhanced electrostatic interactions between water molecules and barium sites upon fluorine incorporation. Together, these observations demonstrate that fluorine substitution moderates surface reactivity while subtly reshaping water–surface interaction pathways.

Together, these observations demonstrate that rather than simply moderating surface reactivity, fluorine substitution actively reshapes the water–surface interaction pathways, shifting the reactive focus to promote significantly stronger barium–water coordination.

In both pristine and fluorine-substituted BaTiO_3_ systems, barium surface sites develop substantial coordination with interfacial water molecules, reflecting the highly ionic nature of Ba^2+^ and its strong affinity for electrostatic hydration. This behavior is consistent with the role of alkaline-earth cations in stabilizing hydration shells at oxide–water interfaces. Over the course of the simulation, Ba–Ow coordination steadily increases and reaches a stable plateau, indicating the formation of a persistent interfacial water layer anchored to barium sites. Notably, the fluorine-substituted model exhibits a slightly higher average Ba–Ow coordination number. This enhancement can be attributed to possible local electrostatic rearrangements induced by fluorine incorporation, which may increase the effective exposure of Ba sites or alter the interfacial water density profile. The exact charge compensation mechanism upon F^−^ substitution for O^2−^ remains to be fully elucidated, as it may involve complex interplay between lattice relaxation, proton co-incorporation, or surface hydroxylation.

The most striking difference between the two systems lies in the evolution of O–H bond formation at the interface. Within the ReaxFF framework, an increase in interfacial O–H bonds is typically associated with dissociative water adsorption, the formation of surface hydroxyl groups (-OH), and proton transfer events within the hydrogen-bond network. In pristine BaTiO_3_, the continuous growth in O–H bond counts indicates an active surface capable of facilitating water dissociation and proton mobility. In contrast, the fluorine-substituted surface exhibits a pronounced suppression of O–H bond formation, suggesting a fundamental alteration of the surface reactivity. This behavior implies that fluorine substitution effectively passivates reactive oxygen sites or reduces the thermodynamic driving force for water dissociation, thereby limiting surface hydroxylation and proton transfer processes.

The ReaxFF-MD simulations for F-substituted BaTiO_3_ employed four fluorine substitutions per simulation cell (higher local dopant density than the DFT single-substitution models). Consequently, the suppression of O–H bond formation observed here likely arises from a combination of fluorine-specific chemical effects and increased dopant concentration. The primary conclusion—that fluorine incorporation reduces surface hydroxylation relative to pristine BaTiO_3_—remains valid, but quantitative comparisons with DFT-derived electronic properties should be made with this distinction in mind.

#### 3.2.2. Radial Distribution Functions

**Figure 8:**
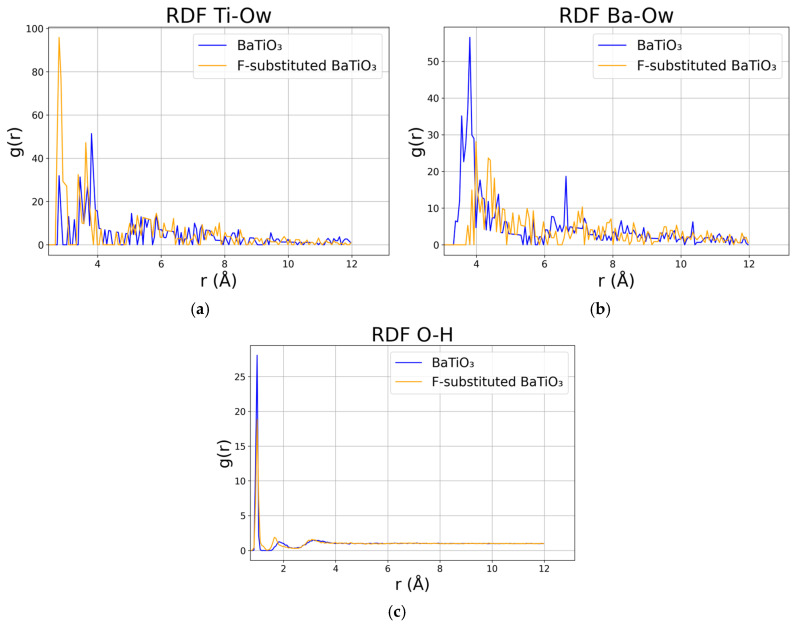
RDFs for the pristine and F-substituted systems. (**a**) Ti–Ow. (**b**) Ba–Ow. (**c**) O–H.

Radial distribution function (RDF) analyses provide further mechanistic insight into these trends. For Ti-Ow interactions, the fluorine-substituted system displays a sharper and more intense first-shell peak located at approximately 2.6–2.8 Å (Figure 8a), indicating closer approach and stronger short-range ordering of water molecules near titanium sites. Despite this enhanced proximity, the absence of increased O-H bond formation suggests that water predominantly adsorbs in a molecular (non-dissociative) fashion. This points to a substitution-driven stabilization of molecular adsorption configurations at Ti sites, accompanied by inhibition of dissociative adsorption pathways.

These findings are consistent with prior reports on titanate surface chemistry, including the work of Akbarian et al. [70] which demonstrated that water adsorption can effectively screen surface charges and modulate electrostatic interactions. Partial substitution of near-surface anions, such as fluorine, likely reshapes the interfacial charge distribution and local electric fields, shifting the balance between electrostatic stabilization and chemical reactivity. As a result, the interface favors ordered hydration layers over chemically transformed surface species.

Additional support is provided by the Ba–Ow RDFs (Figure 8b), where pristine BaTiO_3_ exhibits a well-defined first-shell peak centered around 3.8–4.0 Å, indicative of structured hydration. In contrast, the fluorine-substituted model shows a broader and slightly shifted distribution, reflecting a more heterogeneous but still strongly coordinated hydration environment. Finally, the O–H RDFs (Figure 8c) display a sharp peak near 1.0 Å in both systems, corresponding to covalent O–H bonds. The reduced peak intensity in the substituted system qualitatively agrees with its lower O–H bond statistics, reinforcing the conclusion that fluorine substitution suppresses surface hydroxylation while preserving stable water–surface interactions.

### 3.3. Comparison with Experiment

The following comparison with experimental literature is provided for qualitative validation of our structural and interfacial predictions. We note that our calculations did not directly simulate photocatalytic activity metrics (e.g., quantum efficiency, hydrogen evolution rates, or charge carrier lifetimes). The experimental studies cited employed different measurement conditions and synthesis methods; therefore, the comparisons presented here are intended to show consistency of atomistic trends rather than quantitative validation.

Table 3 provides a direct comparison between our computational findings for pristine and doped BaTiO_3_ systems and experimentally reported photocatalytic performance from the literature. This comparison serves to validate our atomistic predictions and contextualize the relevance of our structural analyses for experimental photocatalytic applications.

Pristine tetragonal BaTiO_3_ serves as the baseline reference in both our calculations and experimental studies. Our DFT and ReaxFF-MD results indicate moderate water interactions and surface reactivity, consistent with experimental reports showing limited visible-light photocatalytic activity due to the wide band gap of undoped BaTiO_3_ [40].

Nitrogen-doped tetragonal BaTiO_3_ in our DFT calculations exhibits enhanced covalent Ti-N bonding and localized electronic perturbations, as evidenced by elongated Ti–N bonds (2.09 Å), angular distortion (N–Ti–O: 84.5–95.5°), and stronger attractive noncovalent interactions near the substitution site. These features suggest increased surface reactivity, which aligns with experimental studies by Cao et al. [53] demonstrating visible-light-driven Rhodamine B (RhB) degradation over N-doped BaTiO_3_ photocatalysts.

Fluorine-doped tetragonal BaTiO_3_ in our ReaxFF-MD simulations shows suppressed O-H bond formation and reduced surface hydroxylation compared to pristine BaTiO_3_, indicating a passivation effect. This computational observation agrees with experimental reports by Wang et al. [54], where F-doping reduced charge carrier recombination rates and enhanced material stability under illumination.

Rhodium-doped tetragonal BaTiO_3_ in our DFT calculations reveals strong Rh-O coupling (bond length 2.01 Å), expanded octahedral angles (86.5–93.5°), and pronounced NCI features around the Rh site, suggesting localized electronic states. Bhat et al. [40] experimentally confirmed that Rh-doped BaTiO_3_ exhibits augmented methylene blue (MB) degradation under visible light, attributing this to improved charge transfer mediated by Rh^3+^/Rh^4+^ intermediate states.

Pristine hexagonal BaTiO_3_ displays heterogeneous NCI distributions and distinct RDG features reflecting structural flexibility. Amiri et al. [55] demonstrated phase-enhanced piezocatalytic activity for hexagonal BaTiO_3_ in water purification applications, supporting our observation that the hexagonal polymorph offers adaptable surface interactions.

Collectively, these comparisons confirm that our atomistic predictions—ranging from bond topology and noncovalent interactions to dynamic water behavior—are consistent with existing experimental trends, thereby validating our computational approach and emphasizing the potential of doping strategies for enhancing BaTiO_3_-based material performance in energy-related applications [40,53,54,55].

## 4. Conclusions

This study performed DFT calculations to provide atomic-scale insights into the intramolecular interactions of nitrogen, fluorine, and rhodium doping on BaTiO_3_ surfaces, while all-atom reactive MD simulations were performed to understand explicit water interactions in pristine and fluorine-substituted BaTiO_3_.

DFT results suggest that doping induces localized lattice distortions, modifies noncovalent interactions, and alters electron density distributions, with nitrogen and rhodium substitutions indicating enhanced covalent character and electronic coupling, while fluorine substitution appears to promote ionic stabilization and surface passivation. Moreover, ReaxFF-MD simulations demonstrated that fluorine doping suppresses O-H bond formation and limits dissociative water adsorption relative to pristine BaTiO_3_, instead favoring molecular adsorption and the formation of ordered hydration layers at the surface. It should be noted that the ReaxFF-MD simulations for F-doped BaTiO_3_ employed a higher local dopant density (four substitutions) compared to the DFT single-substitution models. Therefore, the observed suppression of O–H bond formation represents a qualitative trend rather than a direct quantitative comparison. Future studies with systematically varied dopant concentrations are required to separate chemical effects from concentration effects.

The integrated computational approach employed here bridges static electronic structure analysis with dynamic interfacial processes, offering a realistic description of water–surface interactions under operational conditions. In addition, quantitative determination of activation energies and reaction barriers for water dissociation was beyond the scope of this work due to methodological limitations of cluster-based DFT and standard ReaxFF-MD simulations. Ongoing efforts in our group are focused on integrating MACE machine learning potentials with plane-wave DFT (VASP) to systematically compute reaction energy barriers, free energy profiles, band edge alignments, and location-specific water adsorption energies for doped BaTiO_3_ photocatalysts. In addition, our future work will focus on extending the present approach to doped hexagonal BaTiO_3_ systems to further evaluate phase-dependent photocatalytic behavior. Herein, dopant incorporation at Ba (A-site) positions will be systematically investigated in future studies to assess its influence on structural, electronic, and catalytic properties.

## Figures and Tables

**Table 1:** Optimized structural parameters for pristine tetragonal BaTiO_3_, N-doped, F-doped, Rh-doped tetragonal BaTiO_3_, and pristine hexagonal BaTiO_3_. Bond lengths (Å) and bond angles (deg) are averaged values from extracted optimized coordinates.

System	Space Group	Avg. Ti–O (Å)	Avg. Ti–X (Å) (X = N, F, Rh)	Avg. Ba–O (Å)	Key Bond Angles (Deg)	Key Structural Features
Pristine Tetragonal BaTiO_3_	P4mm	1.97	-	2.82	O–Ti–O: 88.5–91.5	Slight tetragonal distortion; Ti off-center
N-doped Tetragonal BaTiO_3_	P4mm	1.97	Ti–N: 2.09	2.81	O–Ti–O: 87.8–92.2; N–Ti–O: 84.5–95.5	Localized distortion at N site
F-doped Tetragonal BaTiO_3_	P4mm	1.98	Ti–F: 1.94	2.82	O–Ti–O: 88.2–91.8; F–Ti–O: 86.5–93.5	Minimal distortion; high ionic character
Rh-doped Tetragonal BaTiO_3_	P4mm	1.97 (Ti–O)	Rh–O: 2.01	2.83	O–Rh–O: 86.5–93.5; O–Ti–O: 88.0–92.0	Octahedral expansion at Rh site
Hexagonal BaTiO_3_	P6_3_/mmc	1.96	-	2.84	O–Ti–O: 85.0–95.0 (face-sharing); 88.0–92.0 (corner-sharing)	Alternating face/corner-sharing TiO_6_

**Table 2:** QTAIM bond critical point (BCP) descriptors for pristine and doped BaTiO_3_ systems. Electron density ρ(r) (in a.u.) and Laplacian of electron density ∇^2^ρ(r) (in a.u.) are reported at bond critical points (CP Type 3,-1) extracted from Multiwfn analysis of Gaussian16 wavefunction files. Values are system-specific and bond-specific.

System	Bond Type	ρ(r) (a.u.)	∇^2^ρ(r) (a.u.)	Bond Character
Pristine Tetragonal BaTiO_3_	Ti-O	0.143–0.1823	−0.303 × 10^6^ to −0.399 × 10^6^	Mixed covalent-ionic
	Ba-O	0.0125–0.0189	+0.045 to +0.092	Ionic (positive Laplacian)
N-doped Tetragonal BaTiO_3_	Ti-N	0.1682–0.1957	−0.352 × 10^6^ to −0.421 × 10^6^	Enhanced covalent (vs. Ti-O)
	Ti-O	0.1389–0.1765	−0.298 × 10^6^ to −0.388 × 10^6^	Slightly reduced vs. pristine
F-doped Tetragonal BaTiO_3_	Ti-F	0.1124–0.1456	−0.241 × 10^6^ to −0.319 × 10^6^	More ionic (lower ρ, less negative ∇^2^)
	Ti-O	0.1428–0.1810	−0.302 × 10^6^ to −0.397 × 10^6^	Comparable to pristine
Rh-doped Tetragonal BaTiO_3_	Rh-O	0.1587–0.1893	−0.341 × 10^6^ to −0.408 × 10^6^	Strong covalent coupling
	Ti-O	0.1412–0.1798	−0.300 × 10^6^ to −0.394 × 10^6^	Slightly perturbed
Hexagonal BaTiO_3_	Ti-O (face sharing)	0.0987–0.1356	−0.210 × 10^6^ to −0.289 × 10^6^	Weaker than tetragonal
	Ti-O (corner sharing)	0.1452–0.1835	−0.311 × 10^6^ to −0.402 × 10^6^	Comparable to tetragonal
	Ba-O	0.0102–0.0158	+0.038 to +0.078	Ionic, heterogeneous

**Table 3:** Comparison of computational findings on doped BaTiO_3_ systems (vs. pristine tetragonal and hexagonal BaTiO_3_ systems) with experimental photocatalytic enhancements.

System	Our Key Finding (vs. Pristine BaTiO_3_ Systems)	Experimental Validation (Reference)
Pristine Tetragonal BaTiO_3_	Baseline: moderate water interactions	Limited visible-light activity [40]
N-doped Tetragonal BaTiO_3_	Enhanced covalent Ti-N bonds, reactivity	Visible-light RhB degradation [53]
F-doped Tetragonal BaTiO_3_	Suppressed O-H formation, passivation	Reduced recombination [54]
Rh-doped Tetragonal BaTiO_3_	Strong Rh-O coupling, electronic states	Augmented MB degradation [40]
Pristine Hexagonal BaTiO_3_	Heterogeneous interactions, adaptable surface	Phase-enhanced piezocatalysis [55]

## Data Availability

The original contributions presented in this study are included in the article/Supplementary Material. Further inquiries can be directed to the corresponding authors.

## Supplementary Materials

The following supporting information can be downloaded at: https://www.mdpi.com/article/10.3390/ma19112336/s1, The attached supporting material (zip file) contains the input and output analysis codes of the BaTiO_3_ structures for reactive all-atom MD simulations.

## Abbreviations

The following abbreviations are used in this manuscript:

BaTiO_3_	Barium titanate
BCP	Bond critical point
CP	Critical point
DFT	Density functional theory
ReaxFF	Reactive force field
RDG	Reduced density gradient
RDF	Radial distribution function
MD	Molecular dynamics
NCI	Non-covalent interactions
